# Predictive Value of Blood Parameters and Comorbidities on Three-Month Mortality in Elderly Patients With Hip Fracture

**DOI:** 10.7759/cureus.18634

**Published:** 2021-10-09

**Authors:** Esra Demirel, Ali Şahin

**Affiliations:** 1 Department of Orthopedics and Traumatology, Erzurum Regional Training and Research Hospital, Erzurum, TUR; 2 Department of Orthopedics and Traumatology, Ankara City Hospital, Ankara, TUR

**Keywords:** risk factors, surgical treatment, mortality, elderly patients, hip fracture

## Abstract

Background

Knowing the factors that increase the risk of death in patients with hip fractures will help us to take precautions and intervene when necessary in the pre- and postoperative periods. Therefore, it is important to have inexpensive and practical biomarkers that can predict postoperative complications and mortality. The present study aimed to identify the factors that contribute to early mortality in elderly patients with hip fractures in the first three months after trauma, as well as the parameters that may be determinants of mortality.

Methods

The data of 1,015 patients over 65 years of age with femoral neck and intertrochanteric fractures admitted between January 2009 and January 2020 were retrospectively reviewed. A total of 763 patients who met the inclusion criteria were included in the study. Our study was designed to include 110 (14.4%) patients in Group 1 who were determined to have died within three months after the diagnosis of hip fracture and 653 (85.6%) patients in Group 2 who were determined not to have died within one year after the trauma. Age, gender, comorbid diseases, American Society of Anesthesiologists (ASA) score, type of anesthesia, operation time, type of implant used, time until surgery, and some biochemical blood values were compared between the two groups. Our data were analyzed statistically using the IBM Statistical Product and Service Solutions (SPSS) software for Windows, v. 25.0 (IBM SPSS Statistics for Windows, Armonk, NY).

Results

Of all of the patients, 370 (48.5%) were female and 393 (51.5%) were male. The patients who survived had an average age of 76.08, while the patients who died had an average age of 80.57. The mean age among the groups is significantly higher in patients who died. High creatinine, alanine aminotransferase (ALT), lactate dehydrogenase (LDH), and low albumin values were found to be associated with mortality.

Conclusion

It has been determined that advanced age, delayed operation time, high ASA score, and the number of comorbid diseases are associated with mortality in elderly patients with hip fractures, and biomarkers, such as creatinine, ALT, and LDH, can be used as markers for early mortality. With the increase of studies of similar nature, it will be possible to calculate a systematic risk map for mortality in elderly patients with a proximal femur fracture.

## Introduction

Nearly 1.6 million hip fractures occur worldwide each year [1]. It has been found that the risk of death increases following a hip fracture, with the one-year mortality rate increasing by up to 33% [2-3]. The majority of deaths occur within the first three months of surgery, with a five- to eight-fold increased risk of death reported during this period [4-5]. According to a recent meta-analysis, the risk of mortality is the highest during the first three months after a hip fracture, and men are at a higher risk than women [6].

Knowing what factors increase the risk of death in patients with hip fractures will allow us to take pre- and post-surgery measures, as well as intervene as required. In this regard, it is important to identify low-cost and practical biomarkers that can predict postoperative complications and mortality [7]. Patients with hip fractures who had high or low serum sodium, high serum potassium, and elevated serum creatinine had a higher mortality rate [8-9]. C-reactive protein (CRP), which is widely used to monitor inflammatory conditions or infection, as well as a low preoperative hemoglobin level and lymphocyte count, may be indicators of mortality [10]. It has been reported that hypoalbuminemia, an indicator of malnutrition, influences 30-day mortality, the postoperative complication rate, and hospital stay [11]. It has been reported that biochemical marker levels, such as gamma-glutamyltransferase (GGT), alkaline phosphatase (ALP), alanine aminotransferase (ALT), parathyroid hormone (PTH), 25 hydroxyvitamin D, and bilirubin, can be employed to predict mortality by reflecting liver function [12].

The present study aimed to identify the factors that contribute to early mortality in elderly patients with hip fractures in the first three months after trauma and to determine parameters that can be used as markers for early mortality.

## Materials and methods

Patients

Our study received approval from the Erzurum Regional Training and Research Hospital Clinical Practices Ethics Committee (#2021/07-148). The records of 1,015 patients over the age of 65 who were admitted to our hospital's emergency and orthopedic outpatient clinics between January 2009 and January 2020 with femoral neck and intertrochanteric fractures were retrospectively examined. Seventy-five patients whose follow-up could not be reached or whose records were missing, 32 patients with additional fractures, head trauma, or internal organ injury with major trauma, 23 patients with a history of reoperation due to mechanical complications (such as implant failure and prosthesis dislocation), and 122 patients who were found to have died between three to 12 months after the operation were excluded from the study. In our study, we included 110 (14.4%) patients who died within three months of being diagnosed with a hip fracture and 653 (85.6%) patients who did not die for one year after trauma. Patients who died preoperatively after trauma, intraoperatively, and postoperatively were included in the group of deceased patients.

Data collection

The hospital's data processing automation system was used to evaluate the data of patients with hip fractures. The diagnostic screening program scanned S72.0 and S72.1 ICD10 (International Classification of Diseases, 10th revision) diagnostic codes. Age, gender, comorbid diseases, American Society of Anaesthesiologists (ASA) score, type of anesthesia, operation time, implant type used, and time before surgery were registered. The patients' chronic comorbid conditions were reviewed and recorded in previous polyclinic and service records, medication reports, echocardiography reports, and other imaging test reports. Comorbid diseases were classified into 14 categories based on their prevalence. Atherosclerotic cardiovascular diseases, congestive heart failure, rhythm disorders, and valvular disorders were classified as cardiac diseases. Dementia and Parkinson's disease were classified as cognitive disorders. Given the prevalence, the day of death of deceased patients was recorded on the Central Population Administration System (MERNIS: Merkezî Nüfus İdare Sistemi) death reports. Total bilirubin, lactate dehydrogenase (LDH), GGT, ALT, creatinine, international normalized ratio (INR), albumin levels, and neutrophil-lymphocyte ratio (NLR) were recorded during the first admission to the hospital. Data from patients who died within 90 days of the fracture (Group 1) were compared to those who survived one year (Group 2). The correlation of routine blood tests with mortality and diagnoses of death were also evaluated.

Statistical analysis

Our data were analyzed statistically using the IBM Statistical Product and Service Solutions (SPSS) software for Windows, v. 25.0 (IBM SPSS Statistics for Windows, Armonk, NY). In the descriptive analyses, categorical variables were stated as number (n), percentage (%), continuous variables as mean ± standard deviation (SD), and median (min-max) values. Fisher’s chi-square test, Continuity Correction Chi-Square test, and Pearson Chi-Square test were used for comparison of categorical variables between groups. The conformity of continuous variables to normal distribution was evaluated using visual (histogram and probability graphs) and analytical methods (Kolmogorov-Smirnov/Shapiro-Wilk tests). The Mann-Whitney U-test was used for the comparison of data sets that were not normally distributed, and the Kruskal-Wallis test was applied in the comparison of more than two groups. The established model was tested using logistic regression. To perform logistic regression, a model comprised of dependent and independent variables was developed. The Hosmer and Lemeshow tests were used to assess the model's suitability for binary regression. The coefficients of the model's independent variables, as well as their standard errors, Wald statistics, free degrees, significance levels, and odds ratios, were obtained. A value of p < 0.05 was considered statistically significant.

## Results

In our study, the data of 110 patients who died within three months of sustaining a hip fracture and 653 patients who lived for a year were examined. Of all the patients, 370 of them were female patients (48.5%) and 393 of them were male patients (51.5%). The patients who survived had an average age of 76.08 years, while the patients who died had an average age of 80.57 years. The mean age among the groups is significantly higher in patients who died (p < 0.005). Regarding the time of death, 28 (25.5%) of all deaths occurred before surgery, four (3.6%) occurred during surgery, 62 (56.3%) occurred within the first postoperative month, and 16 (14.6%) of the deaths occurred between one to three months after surgery.

According to the analysis of patients who died by age group, 7.4% of patients aged 65 - 74 years, 13.7% of patients aged 75 - 84, and 21.2% of patients aged 85 and over died. It has been found statistically significant that as age increased, so did mortality rates. Female patients died at a rate of 13.6%, while male patients died at a rate of 15%. It was found that gender did not make a significant difference in death rates. Significant differences were found in the implant used, preoperative ASA score, and the type of anesthesia used between the patients who died and those who survived. The data of the variables are shown in Table 1.

**Table 1:** Distribution of Patients According to Their Sociodemographic Characteristics * indicates statistically significant (p < 0.05) results ASA: American Society of Anesthesiologists

Variables		Survivor Group	Mortality Group	P-value
		n (%)	n (%)	
Age	65 - 74	218 (92.8)	17 (7.2)	< 0.001*
	75 - 84	262 (85.9)	43 (14.1)	
	85 and over	173 (77.6)	50 (22.4)	
Gender	Female	319 (86.2)	51 (13.8)	0.629
	Male	334 (85)	59 (15)	
Implant Type	Proximal Femoral Nail	363 (93.3)	26 (6.7)	< 0.001*
	Cemented Partial Prosthesis	99 (90.8)	10 (9.2)	
	Uncemented Partial Prosthesis	172 (81.1)	40 (18.9)	
	Total Hip Prosthesis	19 (90.5)	2 (9.5)	
ASA Score	1	124 (99.2)	1 (0.8)	< 0.001*
	2	219 (96.5)	8 (3.5)	
	3	255 (85.3)	44 (14.7)	
	4	55 (65.5)	29 (34.5)	
Anesthesia Type	General	181 (92.8)	14 (7.2)	0.040*
	Regional	472 (87.4)	68 (12.6)	

The average duration of time until the operation was 5.4 days (range: 1 - 15) in patients who died, while it was 2.8 days (range: 0 - 15) in patients who survived. The risk of death increased statistically significantly as the time between trauma and operation passed (p = < 0.001). The average duration of the operation was 97.9 minutes (range: 35 - 165) in patients who died, while it was 100 minutes (range: 30 - 210) in patients who survived. There was no significant difference between the groups in terms of operation time (p = 0.933).

There was a statistically significant difference in creatinine levels between the two groups (p < 0.05). The creatinine values of the deceased patients were higher than those of the living patients. There was a statistically significant difference in the albumin values of the patients based on their groups (p < 0.05). The albumin values of the living patients were higher than those of the deceased patients. There was a statistically significant difference in the NLR between patient groups (p < 0.05). The NLR of the deceased patients was greater than that of the living patients. There was a statistically significant difference in the LDH values of the patients based on their groups (p < 0.05). The LDH values of the deceased patients were higher than those of the living patients (Table 2).

**Table 2:** Comparison of Blood Parameters * indicates statistically significant (p < 0.05) results ALT: alanine aminotransferase; GGT: gamma-glutamyl transferase; INR: international normalized ratio; LDH: lactate dehydrogenase; NLR: neutrophil lymphocyte ratio

		Mean (min-max)	Median	Standard Deviation	P-value
Bilirubin	Survivor Group	1.57 (0.15 - 58)	0.80	5.24	0.127
	Mortality Group	1.29 (0.1 - 17.1)	0.86	2.00	
INR	Survivor Group	1.14 (0.87 - 2.31)	1.11	0.20	0.193
	Mortality Group	1.29 (0.86 - 6.92)	1.12	0.79	
Creatinine	Survivor Group	0.97 (0.35 - 6.33)	0.87	0.20	< 0.001*
	Mortality Group	1.5 (0.4 - 7.19)	1.10	1.12	
Albumin	Survivor Group	3.65 (1.4 - 5)	3.61	0.49	< 0.001*
	Mortality Group	3.16 (1.31 - 4.6)	3.25	0.67	
NLR	Survivor Group	7.28 (0.55 - 31.13)	7.60	4.47	0.002*
	Mortality Group	9.53 (1.6 - 35)	7.60	6.45	
LDH	Survivor Group	297 (136 - 833)	280	94.15	< 0.001*
	Mortality Group	554 (136 - 8571)	390	85.91	
ALT	Survivor Group	19.2 (4 - 316)	14	23.54	0.130
	Mortality Group	36.2 (6 - 1144)	16	13.39	
GGT	Survivor Group	30.5 (4 - 213)	23	28.46	0.148
	Mortality Group	44.4 (4 - 359)	23	53.23	

In this study, to determine the relationship between having comorbid diseases and death, we found that patients who died had a higher rate of cardiac diseases, cerebrovascular diseases, cognitive diseases, hematological diseases, diabetes mellitus, respiratory diseases, chronic renal failure, and chronic liver diseases. In terms of peripheral vascular disease, malignancy, hypertension, psychiatric diseases, rheumatological diseases, and other endocrine diseases, no significant differences were found between the groups (Table 3).

**Table 3:** Comparison of Comorbid Diseases by Groups * indicates statistically significant (p < 0.05) results

Comorbid Diseases		Survivor Group	Mortality Group	P-value
		n (%)	n (%)	
Cardiac Diseases	Yes	54 (26.1)	153 (73.9)	< 0.001*
	No	56 (10.1)	500 (89.9)	
Cerebrovascular Diseases	Yes	27 (34.2)	52 (65.8)	< 0.001*
	No	83 (12.1)	601 (87.9)	
Cognitive Diseases	Yes	27 (23.6)	87 (76.3)	0.002*
	No	83 (12.8)	566 (87.2)	
Peripheral Vascular Disease	Yes	7 (10.6)	59 (89.4)	0.356
	No	103 (14.8)	594 (85.2)	
Malignancy	Yes	5 (27.8)	13 (72.2)	0.102
	No	105 (14.1)	640 (85.9)	
Hematological Diseases	Yes	9 (39.1)	14 (60.9)	0.001*
	No	101 (13.6)	639 (86.4)	
Hypertension	Yes	77 (16.2)	397 (83.8)	0.066
	No	33 (11.4)	256 (88.6)	
Diabetes Mellitus	Yes	34 (23.4)	111 (76.6)	0.001*
	No	76 (12.3)	542 (87.7)	
Respiratory Diseases	Yes	52 (25.1)	155 (74.9)	< 0.001*
	No	58 (10.4)	498 (89.6)	
Chronic Renal Failure	Yes	22 (43.1)	29 (56.9)	< 0.001*
	No	88 (12.4)	624 (87.9)	
Chronic Hepatitis	Yes	5 (33.3)	10 (66.7)	0.035*
	No	105 (14)	643 (86)	
Psychiatric Diseases	Yes	12 (17.6)	56 (82.4)	0.427
	No	98 (14.1)	597 (85.9)	
Rheumatological Diseases	Yes	3 (15.8)	16 (84.2)	0.863
	No	107 (14.4)	637 (85.6)	

We found that as the number of comorbid diseases increased, so did the mortality rates. There was no death in patients with no comorbid diseases. However, the mortality rate was 7.6% in patients with one comorbid disease, 16.3% in patients with two comorbid diseases, and 26.8% in patients with three or more comorbid diseases. Mortality and the number of comorbid diseases had a significant relationship (p < 0.05) (Table 4).

**Table 4:** Comparison of the Numbers of Comorbid Diseases in Groups * indicates statistically significant (p < 0.05) results

Number of Comorbid Diseases	Survivor Group	Mortality Group	P-value
	n (%)	n (%)	
0	125 (100)	0 (0)	< 0.001*
1	180 (92.4)	15 (7.6)	
2	184 (83.7)	36 (16.3)	
3 and over	164 (73.2)	60 (26.8)	

According to the regression analysis evaluations of the variables, as the patient's age increased by one year, the risk of dying in the first 90 days increased by 1.070 times; as the number of comorbid diseases increased, the risk of dying in the first 90 days increased by 2.161 times; and as the time until the operation increases by one day, the risk of dying in the first 90 days increased by 1.479 times (Table 5).

**Table 5:** Binary Logistic Regression Analysis Results * indicates statistically significant (p < 0.05) results

	β Coefficient	Standard Error	Wald Statistics	P-value	Exp (β) (odds ratio)
Constant	-10.775	1.540	48.969	< 0.001*	0.000
Age	0.068	0.017	15.959	< 0.001*	1.070
Gender	-0.055	0.270	0.041	0.839	0.947
Number of comorbid diseases	0.771	0.115	45.014	< 0.001*	2.161
Time until the operation	0.391	0.051	59.406	< 0.001*	1.479

## Discussion

Advanced age, male gender, and a high ASA score have all been found to be strong evidence indicators of hip fracture mortality [6, 12]. According to our study, advanced age, a high ASA score, and a longer preoperative period significantly increased the risk of death. We have found that males had a higher mortality rate in the first three months following trauma, but the difference was not statistically significant. Many studies have discovered a link between increased preoperative period of post-trauma surgery and increased mortality [13-14]. In our study, it was found that as preoperative time increased, so did mortality. In our multivariate risk regression model, we found that the number of comorbid diseases was the most important indicator of mortality among the variables of age, gender, preoperative time, and the number of comorbid diseases. It has been widely reported in the literature that a high number of comorbid diseases, particularly those associated with cardiovascular pathologies, increase mortality [3, 5]. 

In a meta-analysis by Li et al., it was reported that there was no significant difference in mortality after cemented and cementless arthroplasty after hip fracture in elderly patients [15]. In our study, we have revealed that the mortality rate after cementless partial hip arthroplasty was significantly higher than the groups who underwent osteosynthesis with cemented partial hip arthroplasty, total hip arthroplasty, and proximal femur nail. We believe that this situation is related to the compulsion of non-cemented options in high-risk patients undergoing arthroplasty in order to avoid the damaging effects of bone cement implantation syndrome.

Patients with high plasma creatinine levels have a 2.5-fold higher mortality rate than those with normal values [16]. Glomerular filtration rate, urea, and creatine levels have been reported to be indicators of mortality in patients with hip fractures in the postoperative period, indicating poor general health or comorbidities, such as heart failure, respiratory failure, skeletal muscle damage, or insufficient intravascular volume [17-18]. Our research revealed a link between three-month mortality and hospitalization creatinine levels in patients with hip fractures. High baseline creatinine levels, as previously reported in the literature, were associated with an increased risk of death.

Increased LDH is recognized as a potential biomarker of outcome associated with organ malperfusion. The risk of 30-day mortality is five times higher in patients with hip fractures who had a preoperative serum lactate level of 3 mmol/L or higher [19]. Serum lactate level has been reported as an evidence indicator of prognosis in major trauma and is used in the follow-up of multiple trauma patients [20]. In elderly patients, the proximal femoral fracture is physiologically equivalent to multiple trauma. As a result, it is expected that serum lactate and LDH levels will increase both after fracture and after surgery [21-22]. According to our study, the mean LDH level was found to be 553.81 in patients who died within three months, and 297.13 in patients who survived. The significant difference suggests that LDH levels may be an important evidence indicator of mortality risk in elderly patients with hip fractures.

It has been demonstrated that preoperative nutritional status affects surgical outcomes in orthopedic and other surgical patients [23-24]. In surgical patients, a lack of protein-energy has been linked to increased infection, bedsores, muscle wasting, poor respiratory function, cardiac muscle hypotrophy, and death. There is no universally agreed-upon definition of protein-energy deficiency. Albumin has been shown to predict in-hospital mortality and one-year mortality after hip fracture [25]. Some researchers have proposed that the discriminative value of any variable in the evaluation of nutrition is weak and that nutritional status can be more accurately reflected by examining more than one nutritional parameter in combination [26]. Koval et al. and Symeonidis and Clark employed a combination of serum albumin and total lymphocyte count, both of which are considered to be independent prognostic factors, and found that low total lymphocyte count and albumin levels were associated with in-hospital three-month and 12-month mortality [27-28]. Serum albumin levels were significantly lower in our group of patients who died than in those who survived. 

In our study, the NLR rate, which was determined using the preoperative complete blood count, was significantly higher in the patients who died versus the patients who survived. Patients with an NLR > 5 on the fifth day after hip fracture surgery have a higher risk of postoperative mortality, cardiovascular complications, and infection [29]. It has been proposed that the neutrophil count rises during the acute inflammatory response and serves as a prognostic factor in cardiovascular, abdominal, and oncological surgery [30].

Fisher et al. argued that GGT and bilirubin levels fluctuate in comorbid diseases, are indicators of oxidative stress, and can be used to predict mortality earlier [31]. Bilirubin has antioxidant and anti-inflammatory properties, and low total bilirubin levels have been linked to an increased risk of death in the elderly [28]. Despite the elderly population's long history of low basal bilirubin levels, acute elevation of bilirubin in acute cardiac events, gastrointestinal, and malignancy surgeries are risky in favor of mortality [32-33]. In a study by Ajja et al., it was reported that serum bilirubin levels could be used as a mortality indicator in acute cardiovascular system and gastrointestinal system pathologies [34]. In our study, we found no significant difference in bilirubin levels between patients who died and those who survived, contrary to the common literature. We believe that this matter requires confirmation. Similar studies will be conducted in the future to help eliminate uncertainty in this subject.

Our study had some limitations. First, due to a lack of records, we had to exclude many patients from the study. Second, since our study was retrospective, we were unable to assess the patients' preoperative cognitive and physical conditions. Despite these limitations, we believe that the large series of 763 patients would make a significant contribution to the literature in terms of assessing the risk factors for early mortality in elderly patients with hip fractures and recognizing biomarkers that can be used as risk markers.

## Conclusions

Our study showed that advanced age, delayed operation time, high ASA score, and the number of comorbid diseases are associated with mortality in elderly patients with hip fractures. Similarly, it has been shown in our study that some biochemical parameters, which are checked in the first post-traumatic evaluation, can be used as biomarkers in determining the risk of mortality in elderly patients. With the increase in more detailed studies on this subject, the combined use of known risk factors and biochemical mortality markers may reveal a more detailed and systematic risk map for patients. This will help surgeons and clinicians to anticipate the difficulties they may encounter in the treatment process and to take action more quickly.
